# Cellular Bioenergetics: Experimental Evidence for Alcohol-induced Adaptations

**DOI:** 10.1093/function/zqac039

**Published:** 2022-08-24

**Authors:** Liz Simon, Patricia E Molina

**Affiliations:** Department of Physiology and Comprehensive Alcohol-HIV/AIDS Research Center, Louisiana State University Health Sciences Center, 1901 Perdido Street, New Orleans, LA 70112, USA; Department of Physiology and Comprehensive Alcohol-HIV/AIDS Research Center, Louisiana State University Health Sciences Center, 1901 Perdido Street, New Orleans, LA 70112, USA

**Keywords:** alcohol, bioenergetics, oxidative phosphorylation, glycolysis, metabolically active tissues

## Abstract

At-risk alcohol use is associated with multisystemic effects and end-organ injury, and significantly contributes to global health burden. Several alcohol-mediated mechanisms have been identified, with bioenergetic maladaptation gaining credence as an underlying pathophysiological mechanism contributing to cellular injury. This evidence-based review focuses on the current knowledge of alcohol-induced bioenergetic adaptations in metabolically active tissues: liver, cardiac and skeletal muscle, pancreas, and brain. Alcohol metabolism itself significantly interferes with bioenergetic pathways in tissues, particularly the liver. Alcohol decreases states of respiration in the electron transport chain, and activity and expression of respiratory complexes, with a net effect to decrease ATP content. In addition, alcohol dysregulates major metabolic pathways, including glycolysis, the tricarboxylic acid cycle, and fatty acid oxidation. These bioenergetic alterations are influenced by alcohol-mediated changes in mitochondrial morphology, biogenesis, and dynamics. The review highlights similarities and differences in bioenergetic adaptations according to tissue type, pattern of (acute vs. chronic) alcohol use, and energy substrate availability. The compromised bioenergetics synergizes with other critical pathophysiological mechanisms, including increased oxidative stress and accelerates cellular dysfunction, promoting senescence, programmed cell death, and end-organ injury.

## Introduction

At-risk alcohol use is the costliest form of substance use, with the global economic cost estimated to be about 1300 International dollars/adult, of which 38% are direct costs, and 60% due to loss of productivity.^1^ At-risk alcohol use significantly decreases life expectancy, is linked to more than 2 million years of potential life lost, and is a factor attributed to 5.3% of all annual deaths.^2^ Alcohol-associated pathophysiology is complex, ranging from the risk of injuries and poisoning resulting from acute intoxication to cumulative organ and tissue injury resulting from chronic at-risk alcohol use, and significant impact on mental health in individuals with alcohol use disorder (AUD).^3–5^ However, the severity and prognosis of alcohol-induced tissue injury varies between individuals and depends on factors such as genetic makeup, metabolism, age, gender, ethnicity, environment, and lifestyle.^4^

At-risk alcohol drinking is defined as consumption of more than 3 or 4 drinks on a given day or more than 7 or 14 drinks a week for women and men, respectively.^6^ Binge drinking, a pattern of alcohol consumption that elevates blood alcohol concentrations to equal or greater than 0.08% (80 mg/dL), generally resulting from consumption of 4–5 alcohol drinks over 2 h is also categorized as high risk.^6^ The percentage of the adult population that engages in at-risk alcohol drinking is not trivial, with approximately 18% of adults worldwide reporting heavy episodic drinking in the past month, and 6.6% of adults in the United States reporting at-risk alcohol use.^7^

Several mechanisms are implicated in alcohol-induced multiorgan injury, including alcohol metabolism, oxidative stress, gut dysbiosis and immune activation, inflammation, necrosis and programmed cell death, extracellular matrix remodeling, and epigenomic adaptations.^8–22^ Recent evidence supports the importance of bioenergetic adaptation as an underlying pathophysiological mechanism shared across tissues affected by alcohol that culminates in metabolic instability and end-organ injury. This evidence-based review focuses exclusively on the current knowledge of alcohol-induced bioenergetic adaptations in metabolically active tissues. For reviews on mechanisms of alcohol-induced tissue injury, please refer to Boyd et al. (2022), Massey et al. (2015), McMahan et al. (2020), Molina et al. (2014), Mandrekar and Szabo (2009), Osna et al. (2022), Simon et al. (2021), and Simon et al. (2022) ^23–30^ For reviews on alcohol-mediated mitochondrial adaptations, please refer to Hoek et al. (2002), Bailey (2003), Garcia-Ruiz et al. (2013), Nassir and Ibdah (2014), Han et al. (2017), Steiner and Lang (2017), Shang et al. (2020), and Hao et al. (2021).^31–38^

## Relevance of Altered Bioenergetics to Alcohol-induced Tissue Injury

Bioenergetics in the strict sense is described as the oxidation reactions that occur together with the passage of electrons through mitochondrial membrane protein complexes and coenzymes and is coupled to ATP synthesis. This chemiosmotic hypothesis of oxidative phosphorylation (OXPHOS) was proposed by Peter Mitchell in 1961.^39^ Bioenergetic and metabolism research has since exponentially grown with advances in state-of-the-art techniques to conduct bioenergetic measurements, and their integration with metabolomic, proteomic, and epigenomic data. This approach has significantly improved our understanding of how bioenergetics mechanistically modulate health and disease. At the core of cellular bioenergetics is the mitochondrion, the cell's powerhouse.

Mammalian cell adaptations to energy resources and demands allowed for the evolution of mitochondria with mitochondrial DNA (mtDNA) retaining core genes controlling energy production. Depending on the cellular demands, each cell has a few hundred to thousands of mtDNA copies.^40^ The cellular energy demands are met through intricate communication between the mitochondria, nucleus, and cytoplasm based on substrate availability, reducing equivalents, and reactive oxygen species (ROS) production.^41^

**Figure 1.**
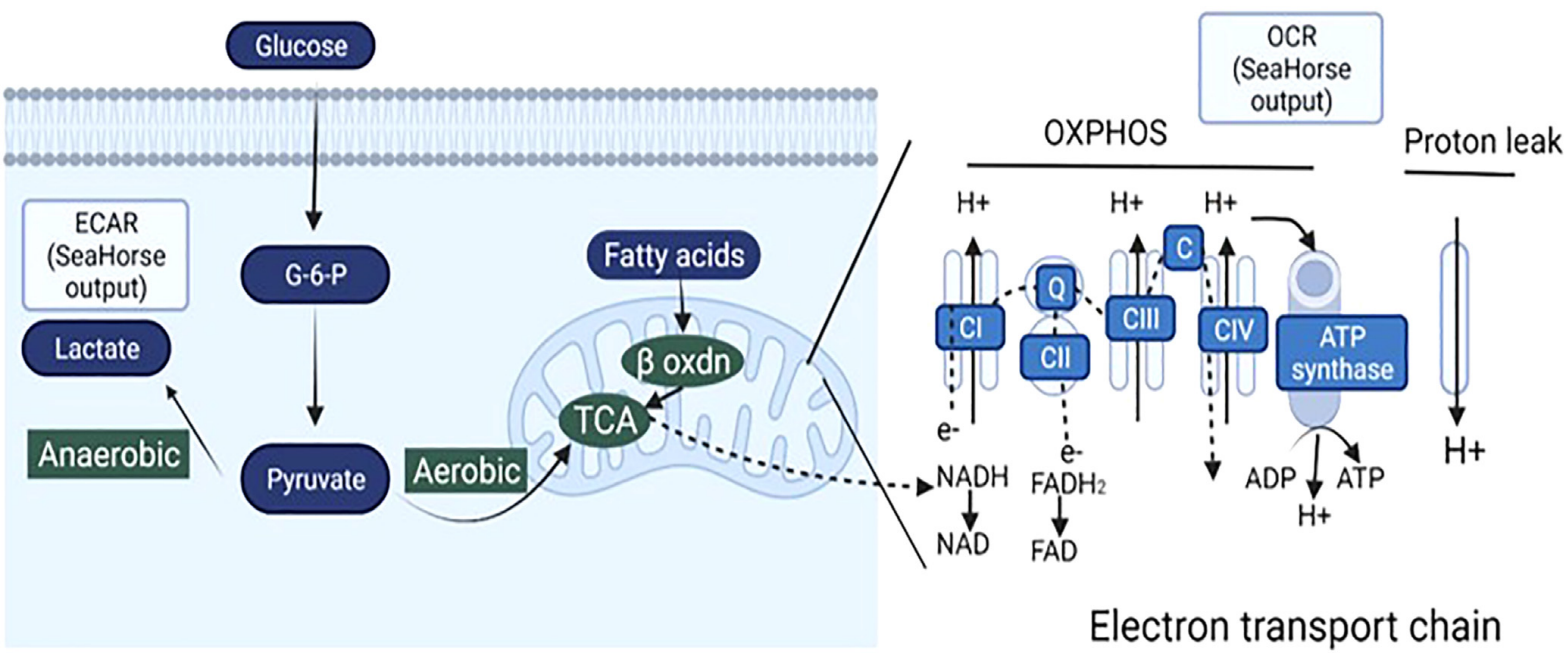
Bioenergetic mechanisms in the cell. Energy substrates are metabolized via glycolysis, TCA cycle and β-oxidation pathways during which electrons (e^−^) are transferred to mitochondrial NAD^+^ and FAD through complex I and II, initiating the ETC. Energy released as e^−^ pass through complexes I, III, and IV generate a trans-inner membrane electrochemical potential. Protons enter the matrix through ATP synthase to generate ATP. OCR can be measured in cells using EFA (Seahorse technology) providing measures of OXPHOS and proton leak. ECAR is measured using EFA indicating lactate produced via anaerobic glycolysis.

Cellular metabolism of energy substrates (glucose, fats, and amino acids) generates ATP needed to meet energy demands and regulates cellular redox state. Glucose is oxidized to pyruvate and in mitochondria containing cells forms acetyl CoA. Fatty acids, on the other hand, are oxidized via the mitochondrial β oxidation pathway to generate acetyl-CoA. Glucogenic amino acids can be converted into glucose, pyruvate or a tricarboxylic acid (TCA) cycle intermediate, and ketogenic amino acids to fat, acetyl CoA or acetoacetyl CoA. The acetyl CoA generated enters the TCA cycle producing Nicotinamide adenine dinucleotide + hydrogen (NADH). The NADH enters the electron transport chain (ETC) and is oxidized by complex I (CI) by transferring electrons (e^−^) to flavin mononucleotide, iron–sulfur clusters (Fe–S), and coenzyme Q, respectively. Succinate, another intermediate of the TCA cycle is oxidized by CII transferring e^−^ to flavin adenine dinucleotide (FAD), Fe–S and coenzyme Q. Cytochrome c reductase (CIII), on the other hand, accepts e^−^ in 2 steps where ubiquinone (CoQ) and ubiquinol (CoQH2) binds to CIII, and this cycle is repeated one more time (Q cycle). The e^−^ are finally transferred to oxygen by CIV (cytochrome c oxidase).^42^ Together with the release of energy, as the e^−^ pass through the redox proton pumps (CI, III, and IV), there is also transport of protons (H^+^) across the mitochondrial inner membrane to generate a trans-inner membrane electrochemical potential. When ATP is low, H^+^ enters the matrix through ATP synthase to generate ATP, which is then exported to the cytoplasm by adenine nucleotide translocators (ANTs).^43^ Because this process of ADP phosphorylation resulting in ATP production requires oxygen, it is known as OXPHOS (Figure 1). This process of mitochondrial ATP synthesis is critically dependent on Ca^2+^ uptake by the mitochondrial calcium uptake complex.^44–48^ Additionally, elegant studies in the Sollott laboratory demonstrated that ATP synthase also serves as the mitochondrial K^+^ transporter and proposed that the transport of both K^+^ and H^+^ is responsible for ATP synthesis.^49,50^ The efficiency with which ETC generates ATP is termed coupling efficiency. In a tightly coupled ETC system, there is maximal ATP production for each calorie used. On the contrary, in a less coupled system, there has to be higher caloric intake for the same amount of ATP to be produced.^41^

**Figure 2.**
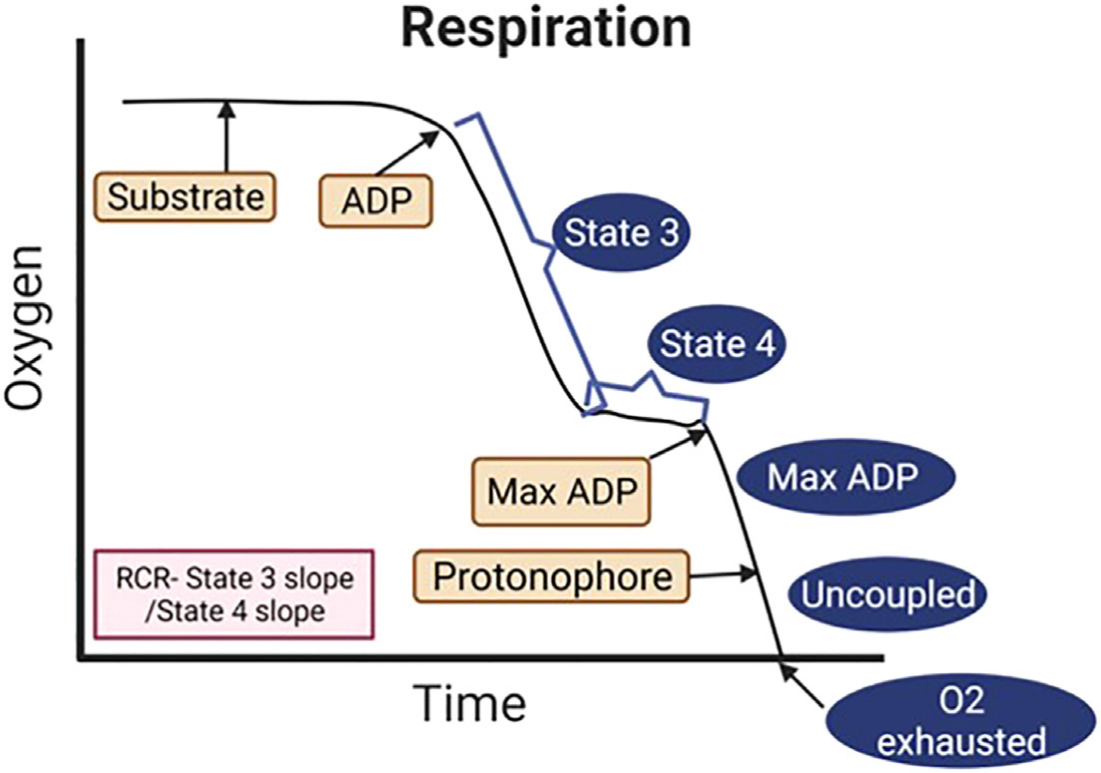
Bioenergetics measured with Clarke electrodes on permeabilized cells or isolated mitochondria. Oxygen consumption is measured in the presence of mitochondrial substrates with or without ADP. State 3 respiration is the maximally activated state and is respiration in the presence of substrates and ADP. State 4 respiration is a resting coupled state and is respiration measured in the presence of substrates alone. Maximal ADP respiration (Max ADP) can be measured if a maximal dose of ADP is added. Uncoupled respiration can be measured after adding a protonophore. RCR is the ratio of respiration state 3/state 4.

Chance and Williams described 5 states of mitochondrial respiration using the oxygraphy protocol (Figure 2).

*State 1* is obtained when the ADP and substrate levels are low, and the respiration rate is slow.
*State 2* is obtained when a high concentration of ADP is added, but the respiration rate is low as substrate levels are low.
*State 3* is induced when substrates are added, and together with the high ADP concentration already available there is low ATP/ADP ratio, and high oxygen consumption (JO_2_).^51^
*State 4* has high ATP/ADP ratio and low ADP levels. The JO_2_ levels are low as oxygen consumption is exclusively from proton leak.^52^
*State 5* indicates ROX or anoxia^53^ . An increase in state 4 respiration or decreased respiratory coupling ratio (ie, state 3 JO_2_/state 4 JO_2_) suggests decreased coupling efficiency.^54^

The redox state is a balance between ROS produced during cellular metabolism, and the antioxidant system that scavenges ROS. Mitochondrial ROS contain unpaired e^−^ and partial reduction of this molecular oxygen produces superoxide (O_2_^•−^) and hydrogen peroxide (H_2_O_2_).^55,56^ O_2_^•−^ and H_2_O_2_ can react with transition metal ions promoting further radical generation, including the highly reactive hydroxyl radical (^•^OH).^41,57^ Moreover, a decrease in the trans-inner membrane electrochemical potential induces mitochondrial permeability transition pore (mtPTP), a self-destructing mechanism, that is activated by increases in mitochondrial matrix Ca^2+^ levels or ROS.^41^ Several antioxidant mechanisms are in place to quench excess ROS generation. Mitochondrial NADPH + H^+^ reduces oxidized glutathione (GSSG) to glutathione (2GSH) using glutathione peroxidases.^41^ Similarly, oxidized thioredoxin-S2 is reduced by NADPH and thioredoxin reductase.^58^ The matrix Mn superoxide dismutase (MnSOD) and Cu/ZnSOD (also present in the inner mitochondrial membrane), convert O_2_**^•−^** to H_2_O_2_. In addition, mitochondrial thioredoxin-2 interacts with mitochondrial peroxiredoxin-3 to decrease ROS.^59^ Thus, as the oxidative/antioxidative balance is tipped from cell's antioxidant defenses to ROS production, damage of lipids, proteins, and nucleic acids lead to oxidative stress, cell injury, and eventual cell death.^34^

**Figure 3.**
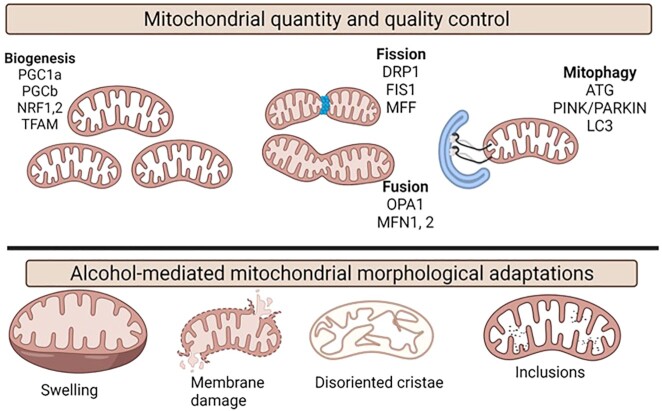
Mitochondrial morphological and functional integrity. Mitochondrial quantity is maintained by biogenesis that is regulated by the major genes, y PPAR γ coactivator 1-/alpha and beta (PGC-1α/b), nuclear respiratory factors (NRF-1 and NRF-2), TFAM. Bioenergetics are closely linked to mitochondrial dynamics that includes fission and fusion events. OPA1 and MFN1 and MFN2 are involved in mitochondrial fusion and mitochondrial fission is regulated by GTPase DRP1, MFF, and fission 1 protein (FIS1). The major mitochondrial repair processes including mitophagy and the major genes implicated are autophagy-related gene (ATG), PINK/PARKIN and microtubule-associated protein 1 light chain 3 (LC3). Some of the major alcohol-mediated changes in mitochondrial morphology include mitochondrial swelling, membrane damage, disoriented cristae, and paracrystalline inclusions.

The overall cellular bioenergetic capacity relies on mitochondrial abundance and quality (Figure 3). Mitochondrial biogenesis allows for increase in size and number of mitochondria and is regulated by peroxisome proliferator-activated receptor (PPAR) γ coactivator 1-/alpha and beta (PGC-1α/b), cotranscriptional factors and its interactions with transcription factors/proteins such as nuclear respiratory factors (NRF-1 and NRF-2), and mitochondrial transcription factor A (TFAM). Together, uncoupling proteins (UCP2), PPARs, thyroid hormone, glucocorticoid, and estrogen and estrogen-related receptors α and γ modulate mitochondrial biogenesis.^60^^61^ Studies indicate that bioenergetics is also intricately linked to mitochondrial dynamics involving fusion and fission events.^62–64^ Optic atrophy protein 1 (OPA1) and mitofusins 1 and 2 (MFN1 and MFN2) are involved in mitochondrial fusion.^65–67^ Mitochondrial fission is regulated by GTPase dynamin related protein 1 (DRP1^68^), mitochondria fission factor (MFF),^69^ and fission 1 protein (FIS1).^70^ In addition, MFN2 is critical for OXPHOS,^71–73^ and decreased MFN1 and OPA1 affect mitochondrial membrane potential, respiration, and reduce the stability of supercomplexes.^74–76^ As discussed, mitochondria are susceptible to ROS, and mitochondrial repair processes including mitophagy are critical in preventing propagation of oxidative stress. Alcohol-mediated effects on mitochondrial biogenesis, dynamics, and mitophagy are reviewed elsewhere.^30,31,34^,^77–81^

## Experimental Assessment of Cellular Bioenergetics

To better interpret the alcohol-induced alterations in cellular bioenergetics, it is important to have a basic understanding of currently available tools. Metabolic flux analysis provides the highest resolution for bioenergetic measurements through the use of isotopomer labeling patterns to measure internal fluxes.^82^ In addition, real-time bioenergetic assays using phosphorescent probes measuring external fluxes, and paired with metabolic substrate and inhibitor protocols allow bioenergetic measurements in short time scales. These popular low molecular resolution assays include respirometry and extracellular flux analysis (EFA).^83^ Respirometry, a potentiometric method that uses platinum and silver electrodes (Clark Oxygraph-2 K; Oroboros) is the most used.^84,85^ The breakthrough Seahorse technology (Agilent Technologies) measures oxygen consumed, and protons released in a very small volume of media just above the cell monolayer (transient microchamber) using sensor probes (www.seahorsebio.com). This also allows for sequential addition of defined metabolic inhibitors to dynamically measure cellular bioenergetics and mitochondrial function.^40,61,86,87^ The disadvantage with most of these systems is that they use extreme assay conditions and may not recapitulate the balance of free energies that are available in vivo.^88^ Here, we briefly discuss the outcome measures using these assays.

*Respirometry using Clark electrodes* is performed using permeabilized cells or isolated mitochondria. Oxygen consumption is monitored in the presence of mitochondrial substrates (glutamate/malate—complex I substrates; succinate—complex II substrates) with or without ADP to measure respiratory states (Figure 2).^89^*OroborosO2k* (*Oroboros Instruments*) is a 2-chamber respirometer with polarographic oxygen sensors allowing for oxygen calibrations and the results integrated using DatLab software. This allows for high resolution respirometry and has evolved over the years allowing for new standards in bioenergetic research. The disadvantage compared to XF96 is that only 2 samples can be run in parallel, but it allows functional examination of the individual mitochondrial respiratory chain complexes.^90,91^

**Figure 4.**
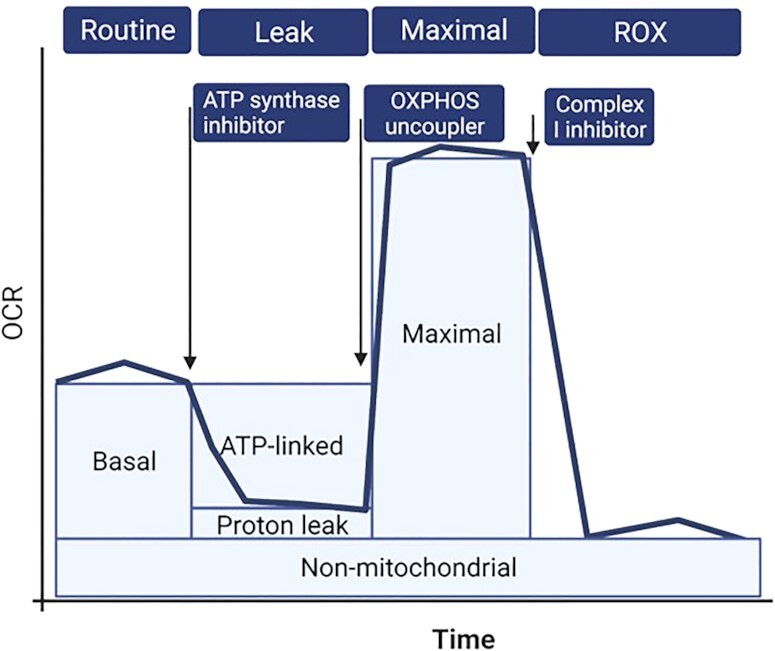
EFA using SeaHorse technology. The Mitostress test is commonly used for bioenergetic measures. It measures basal OCR-baseline oxygen consumption. ATP-linked OCR: basal activity linked to ATP production and the level of proton leak (after injecting ATP synthase inhibitor, oligomycin). Proton-leak OCR: mitochondrial OCR that is not oligomycin-sensitive and shows movement of protons, cations, or substrates across the mitochondrial inner membrane. Maximal OCR: the maximal oxygen respiration (after injecting OXPHOS uncoupler). Nonmitochondrial OCR: ROS generation or other oxygen-consuming processes, including proinflammatory enzymes.

*XF96 EFA*—Intact cells or isolated mitochondria can be used to measure oxygen consumption using the popular Mito stress test. This assay measures (A) basal oxygen consumption rate (OCR): the cell's capacity to meet baseline energy demands and indicates ATP requirement from proton leak. (B) ATP production: this is measured by injecting oligomycin, an ATP synthase inhibitor, and represents basal respiration that drives ATP production. (C) Proton leak: basal respiration that is not coupled to ATP production. An increased proton leak might indicate less efficient mitochondria. (D) Maximal OCR is measured by adding carbonyl cyanide 4-(trifluoromethoxy) phenylhydrazone, a potent uncoupler of mitochondrial OXPHOS. Decreases in OCR reflect deficits in mitochondrial biogenesis, damage to mtDNA or the respiration machinery, or limitations in substrate availability or transport. Other measures include (E) reserve capacity: represents the cell's ability to respond to energy demands and (F) nonmitochondrial OCR: measures nonmitochondrial oxygen consumption after the addition of rotenone (CI inhibitor) and antimycin (CIII inhibitor). Generally, this indicates ROS generation or other oxygen-consuming processes, including proinflammatory enzyme activity. Other outcomes that can be measured are respiratory chain complex activities^40^ and Bioenergetic Health Index (BHI)—log [(reserve capacity × ATP-linked OCR)/nonmitochondrial OCR × proton leak OCR]^92^ (Figure 4). Though SeaHorse technology has allowed for high throughput screening, is user friendly, and allows for measures in small quantities of samples, Oroboros measures are more sensitive and accurate. Nevertheless, significant improvements are being made in both these technologies to mimic physiological conditions and make them robust systems for bioenergetic measurements.^93,94^

Extracellular acidification rate (ECAR), measured either potentiometrically (EFA) or with fluorescent organic cations, is a measure of lactate produced reflecting glycolysis.^95,96^ Other measures include redox potential of NADH using autofluorescence^97,98^; enzyme activity assays^97^; determinations of pH and ion gradients using fluorometric probes^99,100^; and genetically encoded probes to measure changes of intracellular pH, GSH/GSSG ratio, and NAD(P)H redox state.^88^

## Alcohol and Alcohol Metabolites Contribute to Bioenergetic Adaptations

Seminal studies on alcohol metabolism were performed in the early 1900 s. The 1955 Nobel Prize in Medicine was awarded to Dr. Hugo Theorell for his valuable contributions to unraveling alcohol metabolism, including the development of an enzymatic method of alcohol determination. Virtually all tissues metabolize alcohol, with liver accounting for the greatest percentage of alcohol oxidation, posing a major metabolic burden on this organ.^22^ Though alcohol has a caloric value of 7 kcal/g, unlike other energy substrates, alcohol is not stored in tissues and remains in the body water until eliminated. In contrast to other macronutrients, alcohol elimination rate is not hormonally regulated.^22^

Alcohol is metabolized by alcohol dehydrogenase (ADH) mediated conversion to acetaldehyde in the cytoplasm, generating NADH. The reoxidation of the ADH–NADH complex is the rate-limiting step in ethanol oxidation, as the NADH generated must enter mitochondria for oxidation in the ETC.^101^ Because NADH is impermeable, it uses the malate–aspartate or α-glycerophosphate shuttles to enter the mitochondria. Thus, the cellular redox state during alcohol metabolism depends on both the shuttling capacity and efficiency of the ETC. Acetaldehyde is converted to acetate by acetaldehyde dehydrogenase (ALDH) in the mitochondria, also generating NADH and contributing to a decreased NAD^+^/NADH ratio. High acetaldehyde concentrations of 0.6–1.5 m m (reported blood levels are in the µm range^102,103^) are potent inhibitors of the shuttles and the extramitochondrial system that oxidizes NADH is less sensitive to acetaldehyde. Additionally, acetaldehyde inhibits transport of glutamate, phosphate, and citrate that are integral parts of the shuttles, reducing the efficiency of shuttles to transport NADH.^104^ Thus, the overall acetaldehyde-induced inhibition of the NADH shuttles suggests that acetaldehyde production may be a limiting factor in ethanol metabolism.

**Figure 5.**
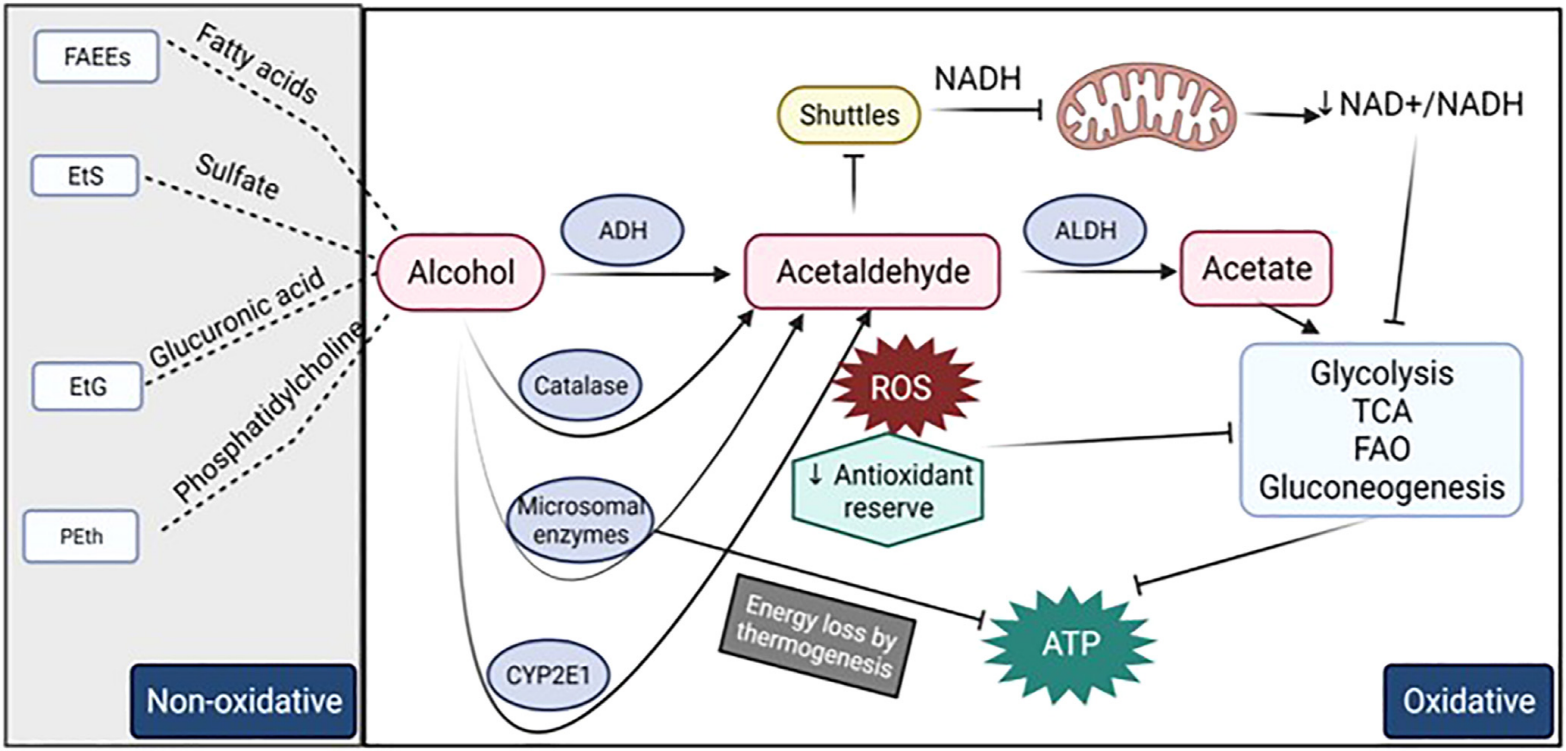
Alcohol metabolism and bioenergetic adaptations. ADH converts alcohol to acetaldehyde in the cytoplasm together with the generation of NADH. NADH is impermeable and uses shuttles to enter the mitochondria. Acetaldehyde is converted to acetate by acetaldehyde dehydrogenase (ALDH), which decreases NAD^+^/NADH ratio. Acetaldehyde is a potent inhibitor of the shuttles. The acetate generated enters metabolic pathways. But the overall decrease in NAD^+^/NADH ratio and the depleted antioxidant levels inhibit key metabolic pathways, including glycolysis, TCA, FAO, and gluconeogenesis resulting in decreased ATP production. Chronic heavy alcohol use induces hepatic cytochrome p450 2E1 (CYP2E1) to metabolize alcohol, which produces high amounts of ROS and impairs antioxidant mechanisms. Microsomal enzymes metabolize alcohol and results in ineffective ATP coupling and net energy loss by thermogenesis. Catalase in peroxisomes also metabolizes alcohol. Alcohol can be metabolized by nonoxidative pathways resulting in enzymatic conjugation to fatty acids, sulfate, glucuronic acid, and phospholipids resulting in the generation of FAEEs, phosphatidylethanol, EtS, and EtG and phosphatidylethanol.

Acetate production from ethanol oxidation that is catalyzed by ADH1 occurs primarily in the liver and accounts for most of the hepatic oxygen consumption.^105^ Acetate can enter metabolic pathways like those used by carbohydrates, fats, and proteins. However, the high NADH levels generated during ethanol oxidation in the liver prevent acetate from entering the TCA cycle because of a reduction of cytoplasmic and intramitochondrial NAD.^106^ NAD^+^ is a coenzyme essential for multiple metabolic pathways, including glycolysis, TCA, OXPHOS, and pyruvate dehydrogenase complex. The enzymes in the hydride transfer catalyzes reduction of NAD^+^ into NADH. Mitochondrial NADH is then utilized by the ETC and is a substrate for ATP production. Thus, an overall decreased NAD^+^/NADH ratio decreases cellular oxidative metabolism.^107^ NAD^+^/NADH ratio also regulates the intracellular redox state, and an increase in NADH generated by alcohol oxidation leads to increased ROS. The decreased NAD^+^/NADH redox ratio and acetaldehyde formation decreases mitochondrial glutathione (mGSH) levels and decreases the antioxidant reserve of the cell. This alcohol-induced decreased mGSH levels are due partly to the impaired transport of GSH through the inner mitochondrial membrane.^108^ Additionally, although chronic ethanol exposure does not alter the cytoplasmic redox ratio, the mitochondrial ratio is decreased.^109^ Ethanol decreases pyruvate levels in the liver^110^ and the resulting decreased pyruvate carboxylase activity decreases gluconeogenesis.^111^ All these metabolic alterations ultimately increase hepatic fatty acid synthesis, which together with increased uptake lead to hepatic lipid accumulation.^111^ Acetate produced by ethanol metabolism can be utilized as energy substrate by tissues, mainly brain, and skeletal and cardiac muscle. Elevated acetate concentrations lead to decreased beta oxidation of long chain fatty acids, the preferred energy substrate in the cardiac and skeletal muscle.^112,113^ In addition, high acetate levels also increase acetylation of proteins, and affect gene expression by increasing histone acetylation that in turn can alter metabolic and other key functional pathways in specific tissues.^111^ Thus, the overall decrease in NAD^+^/NADH ratio and the depleted antioxidant levels inhibit key metabolic pathways, including glycolysis, TCA, fatty acid oxidation (FAO), and gluconeogenesis resulting in decreased ATP production (Figure 5).

Chronic at-risk alcohol use induces additional alcohol metabolic pathways, including hepatic cytochrome p450 2E1 (CYP2E1). Alcohol metabolism through this system produces considerable amounts of ROS and impairs antioxidant mechanisms.^114–116^ In addition, microsomal enzymes are activated in the liver and other tissues,^117,118^ which results in ineffective ATP coupling and net energy loss by thermogenesis.^119^ Some alcohol metabolism occurs by catalase in peroxisomes, though this a minor oxidative pathway. A small fraction of alcohol can be metabolized by nonoxidative pathways resulting in enzymatic conjugation to endogenous metabolites (fatty acids, phospholipids, sulfate, and glucuronic acid) resulting in the generation of fatty acid ethyl esters (FAEEs), phosphatidylethanol, ethyl sulfate (EtS), and ethyl glucuronide (EtG). FAEEs generation is catalyzed by FAEE synthase that is present especially in the brain, heart, liver, and pancreas^120^ (Figure 5).

In summary, alcohol metabolism especially in the liver leads to bioenergetic changes that may potentially contribute to alcohol-induced cellular injury as described in the next section. Alcohol metabolism-mediated oxidative stress is also a major mechanism of alcohol-induced tissue injury, as reviewed elsewhere.^30,34,36,77,121,122^

## Alcohol Modulates Cellular Metabolism

Published evidence of alcohol's effects on energy metabolism dates to mid-20th century. In seminal studies by Ammon and Estler using acute and chronic alcohol administration in mice, decreased ATP content was considered the principal cause for functional cellular damage rather than direct toxic effects of alcohol.^123^ This was later confirmed in preclinical models using chronic ethanol feeding.^124–127^ The implication for alcohol metabolites was supported by studies showing that acetaldehyde in very high concentrations decreases ATP synthesis in hepatic mitochondria.^128,129^

Thus, our understanding of alcohol-induced modulation of cellular metabolism is informed by the data from preclinical models coupled with ex-vivo exposure of cells and tissues to alcohol. The most frequently used chronic alcohol feeding models and their salient features are summarized here. Alcohol self-administration of liquid diets, most frequently the Lieber–DeCarli alcohol diet, providing 100% of daily calories is compared to isocalorically matched control diet.^130^ Other models include a drinking-in-the dark paradigm, where animals have limited periods of access to alcohol in water during the dark cycle for several days or weeks.^131^ Systemic alcohol injections, generally using intraperitoneal injections,^132,133^ or intragastric (IG) infusion through surgically implanted intragastric tubes, (eg, Tsukamoto–French model^134^), or gavages to avoid the influence of taste. In addition, genetically modified mice or rats bred to generate animals with greater or less sensitivity or preference for alcohol have also been used to examine the impact of alcohol on tissue injury.^135,136^ None of these methods provide a perfect reflection of human pattern, quantity, or frequency of alcohol consumption. However, each model provides insight into differential tissue and organ pathophysiological alterations resulting from the different models of alcohol exposure. Moreover, the integration of results from these in vivo studies with those obtained from ex vivo and in vitro ethanol treatment of cells or tissues allow for interrogation of mechanistic hypotheses.

## Alcohol-mediated Bioenergetic Adaptations-evidence From Metabolically Active Tissues Liver

**Figure 6.**
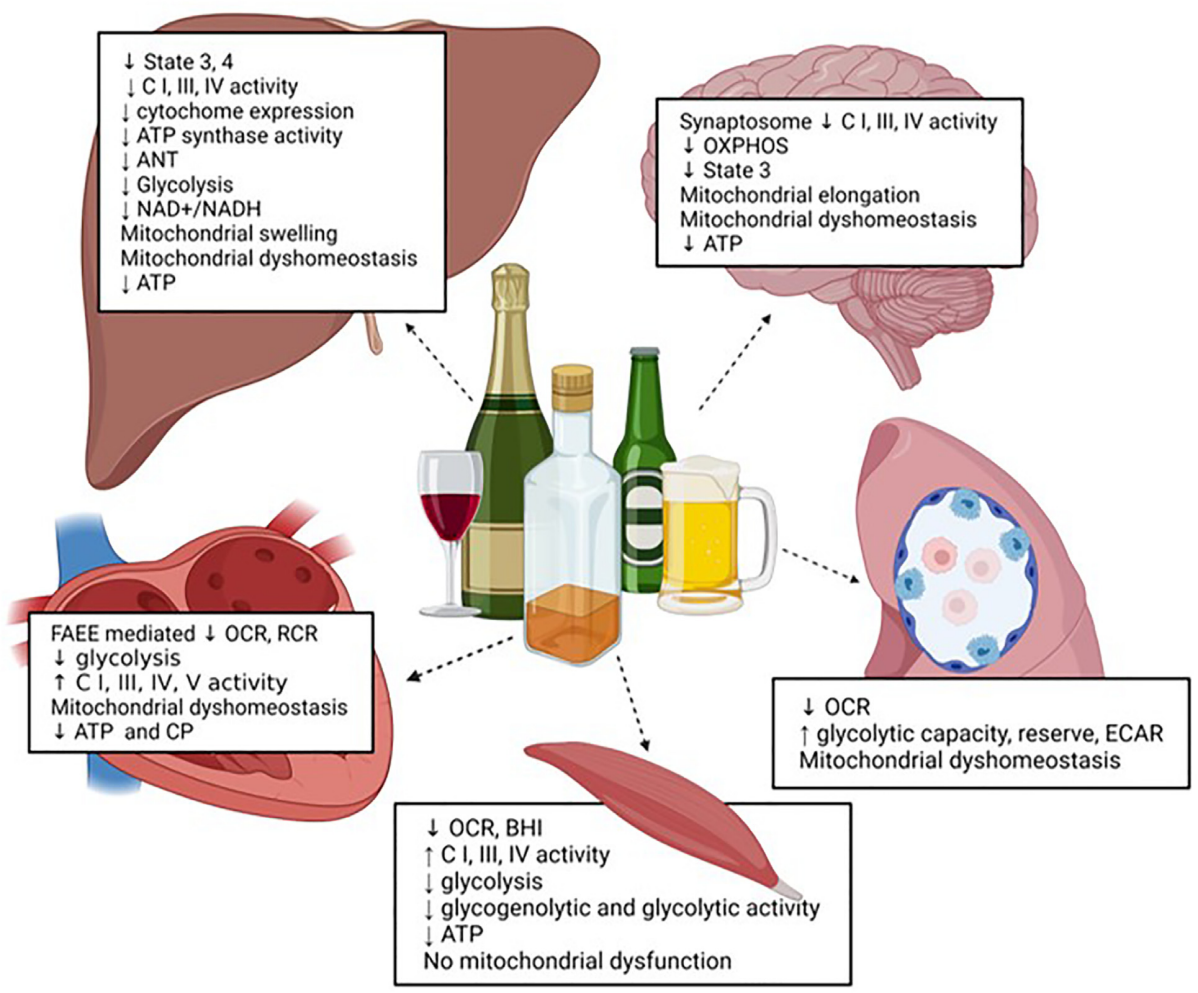
Bioenergetic adaptations in metabolically active tissues. The major alcohol-mediated bioenergetic adaptations in the liver, brain, cardiac and skeletal muscle, and alveolar macrophages.

The liver parenchymal cells are rich in mitochondria and are the main sites for alcohol metabolism. Findings from ex vivo, in vitro, preclinical, and clinical studies have demonstrated that alterations in stages of respiration in the ETC, respiratory complexes, glycolysis, ATP content, mitochondrial morphology, and mtDNA mutations initiate and contribute to the progression of alcohol-related liver disease (Figure 6).

Alcohol, particularly chronic alcohol, exposure decreases bioenergetic respiratory rates in hepatocytes and isolated hepatic mitochondria. Chronic alcohol feeding decreases both coupled and uncoupled respiratory rates, through direct effects on respiration itself, and the coupling efficiency, with a concomitant decrease in ATP synthesis rate.^137,138^ Liver mitochondria isolated from rats administered alcohol in drinking water for 10 wk or fed an alcohol diet had significantly decreased state 4 respiratory rate without significant changes in the proton motive force and OXPHOS at coupling site II. These studies showed that decreased activity of the ETC components, which control coupled but not uncoupled respiration, for example, cytochrome c oxidase activity was affected by alcohol.^139,140^ Liver mitochondria isolated from Lieber–DeCarli alcohol diet fed rats had decreased state 3 respiration rate when multiple substrates were used, and this was associated with decreased CIII and ATP synthase activities.^141,142^ Thayer and Rottenberg also showed that alcohol decreased states 3 and 4 respiratory rates and respiratory control ratio (RCR) in liver mitochondria.^143^ Similarly, liver mitochondria had a significant decrease in state 3 respiration and RCR with increased sensitivity to nitric oxide-dependent inhibition of respiration.^144^ In baboons that were administered alcohol and progressed to fatty liver or advanced fibrosis stages, state 3 respiration was decreased with a decrease in ADP/O ratio and RCR with all substrates. Concomitantly, there was decreased cytochrome expression and glutamate, NADH and succinate dehydrogenase (SDH) activities.^145^ Even moderate alcohol use (10% ethanol for 4 wk) decreased states 3 and 4 respiration with both glutamate–malate and succinate as substrates, decreased cytochrome c oxidase activity and cytochrome aa_3_ content, increased SDH activity, and increased V_max_ of ANT activity in liver mitochondria, suggesting that any amount of alcohol consumption increases bioenergetic burden.^146^ The bioenergetic burden is associated with alcohol-mediated hypoxia and is proposed as a significant factor that contributes to hepatotoxicity and necrosis in both peri-venous and peri-portal hepatocytes. Additionally, hepatocytes from alcohol fed-rats had decreased mitochondrial bioenergetic reserve capacity and greater sensitivity to nitric-oxide dependent inhibition of respiration under both normal and hypoxic conditions providing support to the concept that nitric oxide and alcohol increase susceptibility to hypoxia and exacerbate alcohol-induced hepatic injury.^147^

In addition to alcohol's direct effects on respiration, effects on enzyme expression and activity also contribute significantly to decreased respiration. Chronic alcohol decreases expression of hepatic cytochromes a and b, and cytochrome c oxidase activity.^148,149^ Male rats fed a Lieber–DeCarli alcohol diet had decreased expression of hepatic mitochondrial respiratory complexes I, III, IV, and V. This was also associated with decreased expression of key genes that regulate mitochondrial function, including PGC-1α, NRF1, TFAM, and mtDNA.^150^ In vitro ethanol treatment of hepatocytes for 24 h or isolated mitochondria for 3 h, decreased CI and IV enzyme activity. Ethanol also decreased hepatocyte ADP translocase activity and SDH expression.^151^ As it is known, mitochondrial ETC are organized in supramolecular complexes called respirosomes^152^ that not only increase efficient transfer between complexes but also reduce electron leakage. However, cells expressing mitochondrion targeted CYP2E1 have significantly lower levels of respirosomes, and both in vivo and in vitro alcohol exacerbate the depletion of these respirosomes. Though a major portion of CYP2E1 is in the endoplasmic reticulum (ER), it is significantly expressed in other organelles, including the mitochondria.^153,154^ Mitochondrial CYP2E1 is increased in alcohol-treated animals,^155^ and together with cytochrome c oxidase plays a critical role in alcohol-mediated mitochondrial respiration. Although alcohol significantly decreases mtDNA and mitochondrial genome-encoded mRNA levels, these are potentially secondary effects. Moreover, near complete recovery of respirosomes and DNA damage by mitochondrion-targeted antioxidants and CYP2E1 inhibitors suggest their potential as therapeutic targets to counter alcohol toxicity and tissue damage.^156^ Mitochondrial bioenergetic adaptations are also influenced by alcohol-induced ER stress. Integrating data obtained from activating transcription factor 4 (ATF4) KO mice, alcoholic hepatitis subjects, and liver specific TFAM over expression revealed ATF4 activation to be a key mediator of ER stress that impairs mitochondrial biogenesis and respiratory function through TFAM-mediated pathways.^38^

The route of alcohol administration appears to have subtle differential effects on bioenergetic adaptations of liver mitochondria. For example, rats that self-administer an alcohol liquid diet have decreased glutamate/malate-, acetaldehyde- and succinate-driven state 3 respiration, RCR, and expression of CI, III, IV, and V. However, intragastric (IG) alcohol infusion increased glutamate/malate- and acetaldehyde-driven respiration, NAD^+^/NADH ratio, and decreased succinate-driven respiration.^35^ These differences may be due to different alcohol concentrations achieved with intragastric administration vs. consumption of alcohol in a liquid diet. IG feeding was also associated with increased respiration driven by acetaldehyde, despite decreased complex I and III protein levels. This could be attributed to a phenomenon known as the mitochondrial threshold effect (excess and reserve of mRNA, tRNA, and respiratory chain complexes).^157^ This serves as a protective mechanism especially when there are mtDNA mutations and in the presence of chronic stressors such as alcohol. In addition, differential responses are observed according to species used in experimental alcohol administration. In rats, it appears that there is an excess expression of respiratory proteins, which can account for the discrepancy in the correlation between the levels of respiratory complex proteins and mitochondrial respiration. While in mice, the expression of respiratory proteins is not increased, and there is a strong correlation between mitochondrial respiration and expression of respiratory proteins.^35^ Both alcohol oral self-administration and intragastric infusion increased mitochondrial respiration when glycerol-3-phosphate (which delivers electrons from cytoplasmic NADH to mitochondria) and octanoate (a substrate for beta-oxidation) are used.^35^ Similarly, dietary composition may also modulate bioenergetic adaptations. In rats fed a high-fat, adequate-protein diet, chronic alcohol decreased state 3 and 4 respiratory rates with both malate–glutamate and succinate substrates. Interestingly, these changes were not observed in high-protein low-fat diets. RCR was also significantly reduced with NAD-linked substrates with high-fat adequate-protein but not high-protein low-fat diet.^158^ Finally, substrate availability dictates metabolic function. In the setting of limited pyruvate turnover, alcohol decreases glucose and increases lactate production. However, in the presence of alanine (gluconeogenic precursor), alcohol increases total glucose production in fed animals but decreases production in fasted animals, with a concomitant increase in lactic acid.^159^

Alcohol also mediates glycolytic adaptations, which together with impaired OXPHOS result in a net reduction of ATP and consequent cellular dysfunction. A single oral gavage of 5 g of alcohol/kg body weight decreased ATP generation by glycolysis and maximally increased oxygen uptake in isolated perfused liver 2.5 h after gavage (swift increase in alcohol metabolism).^160^ This decrease is partly contributed by alcohol oxidation itself, especially when malate/aspartate shuttle intermediates are depleted. The reducing equivalents generated during ethanol oxidation compete with that of glycolysis for transfer to the mitochondria and thus inhibit aerobic glycolysis. Simultaneously, there is a decrease in cytoplasmic NAD^+^/NADH ratio that decreases anaerobic glycolysis.^161^ Chronic alcohol interferes with the glycolytic pathway in both normoxic and hypoxic environments in the step between glucose and glyceraldehyde-3-phosphate.^162,163^ And as discussed with OXPHOS, in the perivenous region of the liver, decreased oxygen tension because of ethanol oxidation significantly contributes to decreased glycolytic ATP production thus increasing the risk for liver injury.^164^

Whether mitochondrial morphological changes are the result of bioenergetic adaptations or direct effects of alcohol is not fully understood (Figure 3). Mitochondria are often enlarged, display bizarre shapes, have disoriented cristae, and have paracrystalline inclusions.^165–167^ However, mitochondrial protein content per mtDNA is unchanged and morphological alterations appear to reflect mitochondrial membrane damage rather than an adaptive hypertrophy.^145^ It is suggested that alcohol induces mitochondrial swelling by increasing mitochondrial membrane permeabilization in 2 phases. In the first phase, alcohol instantaneously causes dilution of the whole media and mitochondrial permeability transition allows redistribution of water and alcohol in a concentration dependent manner. In the second phase, there was a time dependent mitochondrial swelling with high ethanol concentrations. Together, the eventual disruption of the K^+^ gradient contributes significantly to mitochondrial swelling.^168^ It is possible that the alcohol-mediated bioenergetic adaptations are mediated by mitochondrial remodeling. Mitochondrial remodeling includes dynamic changes in fission and fusion; mitophagy and biogenesis; and together with calcium signaling, ROS production, and epigenomic alterations.^169,170^ In fact, Han et al., using the intragastric and oral feeding alcohol models, proposed that mitochondrial remodeling is critical for hepatic mitochondrial adaptations driving bioenergetic changes in response to alcohol.^35^ However, though mitochondrial remodeling in the short term can be a compensatory mechanism, the chronic stress on the liver long term can lead to liver injury.

Although alcohol metabolism poses a major metabolic burden on the liver, alcohol-mediated bioenergetic adaptations are also observed in metabolically demanding tissues, including the cardiac and skeletal muscle, pancreas, and brain (Figure 6).

## Cardiac and Skeletal Muscle

Cardiac and skeletal muscle have high metabolic demand and are rich in mitochondria. In the cardiac tissue, mitochondria account for ∼35% of the volume^171^ and at rest generate ∼90% of ATP requirements. In skeletal muscle, mitochondria constitute about 3–8% of the volume,^172^ which is highly influenced by physical activity. Thus, direct, or indirect effects of alcohol-mediated impaired cellular bioenergetics lead to dysfunctional cardiac and skeletal muscle mass.

Chronic administration of alcohol in dogs decreased cardiac intramitochondrial isocitrate dehydrogenase, ATP, mitochondrial oxygen consumption, and RCR leading to altered myocardial performance.^173,174^ Similarly, in rats receiving chronic alcohol there was increased cardiac muscle glucose-6-phosphate dehydrogenase, aldolase, and glyceraldehyde phosphate dehydrogenase activity but decreased isocitrate dehydrogenase activity.^175^ In the heart, FAEEs are the common alcohol metabolites and myocardial mitochondria incubated with FAEEs decreased RCR and maximal OCR. Furthermore, FAEEs bind to mitochondria, and importantly when incubated with ethanol, 72% of intracellularly synthesized ethyl esters bind to mitochondria. Cleavage of FAEEs and generation of fatty acids results in uncoupling of OXPHOS, thus FAEE may act as a toxic shuttle for fatty acids.^176^ Acute alcohol also affects cardiac cell bioenergetics. Intraperitoneal alcohol injection results in a significant increase in glycolysis and decreased mitochondrial respiration and RCR within 30 min, which disappear with decreasing blood and myocardium alcohol levels. However, chronic intraperitoneal injection of the same amount of alcohol decreased glycolysis, glycogen content, mitochondrial respiration, and RCR of isolated cardiac mitochondria with decreased ATP and creatine phosphate.^177^ Thus, it appears that acute alcohol may elicit a compensatory increase in glycolysis to meet the energy demands. In contrast, chronic alcohol administration decreases both glycolytic and mitochondrial respiration potentially leading to significant decreases in ATP production and thus impaired cardiac function. Chronic alcohol also increases cardiac expression of PPARα and decreases PGC-1α indicating impaired cardiac fatty acid metabolism. This was also associated with decreased mRNA, protein, and GAPDH activity indicating impaired glycolytic energy production. In addition, there was an increase in fructose content indicating a compensatory adaptation for activation of alternate glucose metabolism pathways, such as the sorbitol pathway to meet energy demands.^178^ Contrary to what is observed in the liver, there is a significant increase in mitochondrial enzyme activities (citrate synthase, CI, III, IV, V), and adaptive increase in mtDNA indicative of increased mitochondrial number in the cardiac tissue from alcohol-fed animals. These results suggest tissue-specific bioenergetic adaptations in response to alcohol.^179^

Functionally, skeletal-muscle mitochondria have a high RCR, ADP/O ratio and a high state-3 respiration rate with different substrates. However, the adverse effects of chronic at-risk alcohol use on skeletal muscle mitochondrial function are unclear. Using unbiased microarray analysis of myotubes treated with 100 m m ethanol, defects in ETC components, endogenous antioxidants, and enzymes regulating TCA cycle were identified to be differentially regulated. In vitro ethanol also impaired cellular respiration, decreased function of complexes I, II, and IV, and reduced OXPHOS. This decreased ATP content and redox ratio dysregulated succinate oxidation in the TCA cycle.^180^ However, Cardellach et al.^181^ argue that alcohol-related myopathy is not associated with decreased mitochondrial energy supply. Membrane preparations from liver and skeletal muscle from the same alcohol-fed animals showed that liver membranes developed membrane tolerance while muscle membrane retained normal sensitivity to alcohol effects. The authors conclude that the lack of development of muscle membrane tolerance correlates with lack of chemical changes in phospholipids, normal function of mitochondria and sarcoplasmic reticulum, and that there is not a deficiency in mitochondrial energy supply in the skeletal muscle.^181^ Using skeletal muscle biopsies from people with at-risk alcohol use, the oxidation rates with different substrates, the activity of respiratory chain complexes, and the cytochrome content were unaffected.^182^ Similarly, Trounce and co-workers reported normal muscle glycogen, carnitine levels, and normal activities of mitochondrial marker enzymes in people with chronic at-risk alcohol use.^183^ However, they attributed decreased glycogenolytic and glycolytic enzyme activity to the observed type 2 fiber atrophy that is commonly seen with chronic at-risk alcohol use.^182,183^ Contrary to this argument, cytochrome c oxidase activity and mitochondrial volumes were lower with higher creatine phosphate content in muscle of people with at-risk alcohol use.^184^ Additionally, sharing mitochondrial contents by fission–fusion events was decreased by both in vivo genetic perturbations and chronic at-risk alcohol use. A mitofusin 1-dependent pathway was identified, where inhibiting fusion reduced the mitochondrial metabolic reserve and dysregulated calcium oscillations during prolonged stimulation. Thus, prolonged loss of fusion jeopardizes bioenergetics and excitation–contraction coupling, providing a potential mechanism contributing to alcohol-related myopathy.^185^ Thus, although there are suggestions that at-risk alcohol use may not affect mitochondrial energy supply, alcohol-mediated impairment of bioenergetics may significantly contribute to exercise intolerance, metabolic dyshomeostasis, and impaired regenerative capacity of muscle stem cells.

Work from our group demonstrated that chronic binge alcohol (CBA)-induces skeletal muscle mitochondrial gene dysregulation at end-stage disease of simian immunodeficiency virus (SIV) infection in antiretroviral therapy (ART) naive rhesus macaques. CBA and ART decreased SDH activity in type 1 and type 2b fibers and gene expression of PGC-1β in the skeletal muscle of CBA/SIV macaques compared to uninfected controls. Moreover, the SIV infection-mediated upregulation of mitophagy-related gene expression was prevented by CBA. These findings suggest that SIV infection disrupts mitochondrial homeostasis and combined with CBA, results in differential expression of genes involved in mitochondrial dyshomeostasis.^186^ Moreover, myoblasts isolated from CBA/SIV/ART+ macaques showed decreased maximal OCR and formoterol, a β adrenergic agonist, partially restored maximal OCR.^187^ Results from our preclinical studies have now been translated to the study of dysglycemic people living with HIV (PLWH) and at-risk alcohol use. Our data show that a higher Alcohol Use Disorders Identification Test (AUDIT) score was associated with negative indicators of bioenergetic health, including proton leak, nonmitochondrial oxygen consumption, and BHI in skeletal muscle of PLWH. This was also associated with increased mitochondrial volume and decreased expression of genes implicated in mitochondrial function.^188^ Apart from how bioenergetic changes can regulate skeletal muscle metabolic capacity, we previously published evidence that alcohol-mediated shifts in bioenergetic phenotype underlie impaired differentiation of muscle stem cells. In vitro ethanol treatment of primary macaque myoblasts increased myoblast maximal OCR and decreased glycolytic metabolism (ECAR) at D0 of differentiation, which was associated with ethanol-mediated decreases in fusion index and myotubes per field, indices of myoblast differentiation. Moreover, alcohol impaired myoblast glycolytic metabolism, which may negatively impact the ability of myoblasts to fuse during muscle regeneration in vivo.^189^

## Pancreas and Adipose Tissue

Alcohol-mediated impaired bioenergetic function is seen in alcohol-related pancreatitis. In fact, bioenergetic dysfunction is postulated to be one of the etiopathologies of alcohol-related pancreatitis. Using primary mice and human pancreatic acinar cells or pancreatic acinar AR42J cell line treated with ethanol, acetaldehyde, FAEE, or their combinations, it has been shown that ethanol significantly decreased total ATP production and increased mitochondrial stress.^190–193^ Acetaldehyde increased ATP from glycolysis but inhibited mitochondrial ATP turnover suggesting a compensatory metabolic adaptation when OXPHOS is impaired. However, FAEE inhibited both glycolytic and mitochondrial ATP production suggesting that alcohol exacerbated the inability of cells to meet cellular energy demands leading to acinar cell dysfunction and apoptosis. In addition, both acetaldehyde and FAEEs decreased spare respiratory capacity and impaired mitochondrial reserve.^190^ Oxidative ethanol metabolism activating MPTP and mitochondrial failure, is also proposed as a mechanism of decreased ATP production in pancreatic acinar cells.^194,191^ In particular, the ethanol or FAEE-mediated increased Ca^2+^ has been identified as a major contributor to the observed decrease in ATP production, increased trypsinogen activation, and cell death associated with acute alcohol-related pancreatitis.^191^

Clinical and preclinical studies show that alcohol decreases circulating basal insulin levels^195,196^ and circulating insulin and C-peptide expression in response to glucose.^197^ Using the Frequently Sampled Intravenous Glucose Tolerance Test, studies from our group have shown that CBA significantly impairs endocrine pancreatic response to a glucose load in SIV-infected macaques.^198,199^ Moreover, in vitro alcohol exposure decreases glucose-stimulated insulin secretion from human^200^ and rodent pancreatic islets^196,201,202^ and increases β-cell apoptosis.^203–205^ However, the mechanisms responsible for alcohol-mediated impaired pancreatic endocrine function are largely unknown. In type 2 diabetes, there is decreased oxygen consumption, decreased ATP:ADP ratio, and increased oxidative stress in pancreatic β cells. Because mitochondrial function is critical for glucose stimulated insulin release by β cells, and evidence indicate bioenergetic impairments as a pathomechanism in alcohol-induced pancreatitis, studies are warranted to determine whether alcohol-mediated bioenergetic alterations mechanistically contribute to the observed decreased pancreatic insulin secretion. Similarly, alcohol impairs adipocyte metabolic functions^206–209^ and optimal mitochondrial bioenergetic function is crucial for differentiation, lipogenesis, lipolysis, and secretion of adipokines.^210^ Preclinical and clinical studies in obesity indicate that mitochondrial oxygen consumption, expression of OXPHOS proteins, and mitochondrial biogenesis are reduced in adipose tissue or isolated adipocytes.^211–213^ However, whether alcohol impairs adipocyte bioenergetic function is not known. Thus, although these 2 tissues are critical in controlling whole body energy metabolism, there exists a gap in literature on how alcohol-mediated bioenergetic adaptations alter endocrine pancreatic and adipose function.

## Brain

The bioenergetic adaptations of the brain to alcohol have been studied in efforts to determine their contribution to addiction and neuronal injury. Studies in zebra fish show that acute alcohol increased brain baseline respiration, CI-mediated OXPHOS, coupling efficiency, bioenergetic efficiency, and residual oxygen consumption to electron transfer system (ROX/ETS) ratio. In contrast, chronic alcohol administration decreased baseline respiration, complex I and II-mediated ETC, and increased ROX state and ROX/ETC ratio.^214^ In mice administered alcohol (3.8 g/kg) intraperitoneally and sacrificed after 6 h (modeling alcohol hangover), there was a decrease in malate–glutamate state 3 respiration and ATP production rates, and an increase in synaptosome respiration driving proton leak and spare respiratory capacity. This was also associated with decreased synaptosome CI, II, III, and IV activities demonstrating that alcohol-induced bioenergetic adaptations in the brain are potentially due to mitochondrial functional changes at the level of synapses, and that the decreased motor performance could be associated with brain bioenergetic dysregulation.^215–217^ Similarly, 2 h after an acute intraperitoneal alcohol injection (50 mmol/kg), there was significant inhibition of state 3 respiration in the brain.^218^ In vitro ethanol (50–200 m m) inhibited depolarization mediated OXPHOS stimulation. Mechanistically, it was demonstrated that ethanol inhibited voltage gated Ca^2+^ channels thus inhibiting synaptosomal free Ca^2+^ increase. This was in part by stimulation of the mitochondrial Ca^2+^/Na^+^ antiporter, which inhibited free Ca^2+^ increase in the mitochondrial matrix. The inhibition of the excitation-induced stimulation of synaptic OXPHOS is suggested to contribute to the depressant and narcotic effects of alcohol.^219^ After 8 h of withdrawal from chronic intermittent alcohol exposure, mitochondria were elongated, and mitochondria-on-a-string were observed in medial prefrontal cortex. This was associated with significant reduction in mitochondrial bioenergetics, including ETC, decreased gene expression of Mfn2, and increased fission.^37^ In the brain there were no changes in the substrate shuttles and ethanol oxidation, but there was decreased NAD^+^/NADH ratio and impaired OXPHOS.^220^ Isolated brain mitochondria from chronic alcohol fed-mice had decreased CI and V activity and decreased carnitine palmitoyl transferase 1 (cPT1) and cPT2 levels that are required of acylation of fatty acids from outer to inner mitochondrial membrane for ATP production. This was also associated with decreased β oxidation of palmitate suggesting that impaired substrate entry step (CI function) can affect ATP production (CV function). There was also increased cytochrome c leakage associated with the decreased cPT1/cPT2, while decreased CI and V paralleled a decrease in depolarization of mitochondrial membrane potential and ATP production.^221^ Overall, significant evidence supports neuronal bioenergetic adaptations to acute and chronic alcohol exposure.

Changes in mitochondrial bioenergetics are also observed in pups of rats administered alcohol during pregnancy. Cerebella of pups had significantly reduced mRNA levels of mitochondrial genes encoding CII, IV, and V in cerebellar granule cells. In vitro ethanol treatment of cerebellar cells reduced neuronal expression of mitochondrial genes encoding CIV and V, impaired mitochondrial function, and ATP production. These data strongly suggest that mitochondrial dysfunction may contribute to the underlying pathophysiology of fetal alcohol syndrome.^222^

Several candidate genes are implicated in affecting alcohol-mediated bioenergetic adaptations in the brain. After 1 wk of binge ethanol administration in adolescent rats, it was demonstrated that melanocortin 4 receptor (MC4R) agonist reduces hippocampal oxidative damage by increasing 2 key mitochondrial genes NRF2 and PGC1α, increasing mitochondrial volume, decreasing mitochondrial calcium levels, and increasing respiration complex expression.^223–225^ Using “drinking in the dark” paradigm, it was shown that alcohol-induced hippocampal oxidative damage and astrocyte activation in adolescent mice were associated with decreased MFN2 expression implicating impaired mitohormetic response and bioenergetic function in alcohol-associated neuroinflammation.^226^

Thus, published evidence suggest that alcohol-mediated bioenergetic adaptations are shared across metabolically active tissues leading to pathophysiological alterations (Figure 6). How these bioenergetic changes synergistically interplay with other alcohol-mediated pathomechanisms leading to end organ injury remains to be fully understood and is critical in identifying therapeutic targets to ameliorate disease burden.

## Gaps in Knowledge of Alcohol-mediated Bioenergetic Adaptations and Cellular Function

This review focused on describing alcohol-mediated alterations in bioenergetics of tissues central to homeostasis of whole-body energy metabolism. However, it is well established that one of the mechanisms of chronic at-risk alcohol use is immune activation and inflammation, and an inability of the immune system to respond to infections.^227–229^ Though alcohol-mediated changes in tissue bioenergetics are well characterized, there is a gap in the literature on alcohol-mediated alterations of immunometabolism. Early work from our group showed that alcohol intoxication impairs endotoxin-induced increases in glucose metabolism and attenuates glucose utilization, particularly by tissues rich in immune cells.^230,231^ Increased metabolic rate by cells of the immune system is a required component of an adequate host defense. More recent evidence of perturbed alcohol-mediated immunometabolism comes from studies in alveolar macrophages. Yeligar and colleagues have demonstrated that ethanol-induced oxidative stress via NOX4 impairs alveolar macrophage phagocytic function. This was attributed to decreased basal respiration, ATP-linked respiration, maximal respiration, and spare capacity of alveolar macrophages, all indicative of bioenergetic adaptations. This was also associated with increased glycolytic capacity, glycolytic reserve, and nonglycolytic acidification, with concomitant increases in hypoxia-induced factor 1α expression and activity, phosphorylation of pyruvate dehydrogenase, and extracellular lactate levels in alveolar macrophages.^232^ However, immune cells, particularly CD4^+^ T cells, rely on both glycolysis and OXPHOS for energy requirements for immune responses.^233^ While naïve CD4^+^ T cells rely on β oxidation, when activated they rely on aerobic glycolysis.^233^ The subsequent differentiation depends on distinct metabolic pathways, with Th1, Th2, and Th17 depending on glycolytic pathway, while Treg relies on OXPHOS.^234,235^ Results from recent studies from our group provide evidence for the first time that ethanol decreased maximal respiration, coupling efficiency, OCR-linked ATP production and decreased BHI with simultaneous increases in OCR-linked proton leak in differentiated CD4^+^ T cells. This was also associated with increased glycolysis, which when inhibited prevented the ethanol-mediated increase in Th1 cells without affecting Tregs. In addition, ethanol increased Treg mitochondrial volume and altered expression of genes implicated in mitophagy. Overall, the evidence suggests that ethanol impairs CD4^+^ T cell immunometabolism and mitochondrial repair processes promoting a pro-inflammatory phenotype of CD4^+^ T cells.^236^ Whether this immune cell bioenergetic maladaptation functionally contributes to alterations in adaptive immune responses in people with at-risk alcohol is under active investigation.

## Summary and Perspectives

Alcohol and its metabolites permeate virtually all tissues eliciting cellular stress. State-of-the-art techniques to determine cellular bioenergetic measurements and their integration with omic and targeted approaches have advanced our understanding of alcohol-induced adaptations to meet the energetic demands in response to cellular stress. In metabolically active tissues, alcohol impairs bioenergetic processes and simultaneously increases oxidative stress, thus decreasing metabolic flexibility. Fundamental to this is dysregulated mitochondrial morphological and functional integrity. This alcohol-mediated vicious cycle of compromised bioenergetics and increased oxidative stress can potentially initiate senescence and programmed cell death ultimately leading to tissue injury. Together, these bioenergetic adaptations synergize with other critical pathophysiological mechanisms exacerbating alcohol-induced end-organ injury. There have been significant strides in elucidating bioenergetic adaptations in metabolically active tissues, but the relevance and significance of alcohol's contribution to altered immunometabolism remains to be fully elucidated. This is especially relevant in lieu of the immune response to an infectious or noninfectious challenge, the ability of the immune system to clear bacterial or viral pathogens, and robust responses to vaccines. Advances in the fundamental knowledge of how alcohol elicits cellular injury will provide insight into novel strategies to ameliorate disease burden.

## Funding

This work was supported by the National Institute on Alcohol Abuse and Alcoholism of the National Institutes of Health under award number P60 AA009803. LS and PEM have received grant support from the NIH (P60 AA009803).

## Data Availability

No new data were generated or analyzed in support of this research.
